# Venous Intravasation During Hysterosalpingography

**DOI:** 10.7759/cureus.20489

**Published:** 2021-12-17

**Authors:** Savvas P Deftereos, Vasileios Balomenos, Konstantinos Frigkas, Chrysovalantis Stylianou, Soultana Foutzitzi

**Affiliations:** 1 Radiology, Democritus University of Thrace, Alexandroupolis, GRC; 2 Radiology, University Hospital of Alexandroupolis, Alexandroupolis, GRC; 3 Radiology and Interventional Radiology, University Hospital of Alexandroupolis, Alexandroupolis, GRC; 4 Radiology and Interventional Radiology, Democritus University of Thrace, Alexandroupolis, GRC

**Keywords:** pelvic veins, uterine cavity, hysterosalpingography, intravasation, venous

## Abstract

Hysterosalpingography (HSG) is an imaging method performed to assess tubal occlusion in cases of infertility, ectopic pregnancy, and hyperplasia. Although venous intravasation (VI) is a rare occurrence during HSG, it is associated with thromboembolic episodes and misinterpreted HSG. We present a rare case report of a 41-year-old female who underwent HSG and the introduction of contrast medium to the pelvic drainage system via the uterine cavity and the myometrium.

## Introduction

Hysterosalpingography (HSG) is an imaging method that uses iodinated contrast media and fluoroscopy to examine the uterus and the fallopian tubes. It is most commonly performed in female individuals who have infertility and habitual abortions [1]. In addition, HSG is recommended in cases of hyperplasia, fibroids, ectopic pregnancy, and polyps. It can also help evaluate an obstruction of the fallopian tubes when the latter is caused by scarring, ectopic pregnancy, or recanalization procedures [2, 3].

Venous intravasation (VI) concerns an unintended reverse flow of the injected contrast medium into the adjoining venous system. As a result, the contrast medium flows from the uterine cavity towards the myometrial venous and pelvic veins. VI can be observed either in a linear pattern (multiple thin lines) or reticular pattern, especially in conditions with elevated intrauterine pressure (e.g., forceful contrast-injection, tubal obstruction). Although a rare occurrence (0.4%-6.9%), VI is associated with several complications, with pulmonary embolism being the most severe among them (especially with oil-soluble compared to water-soluble contrast) [4, 5].

Here, we report a case of subtle VI that could be misinterpreted as intraperitoneal spillage from a uterine perforation.

## Case presentation

**Figure 1:**
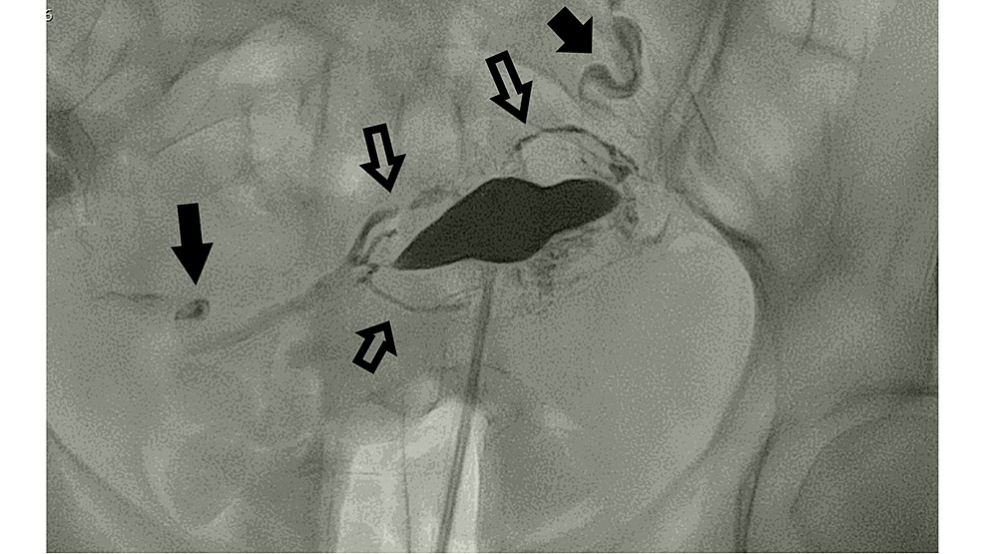
Initial image. Depiction of uterus cavity with myometrial vessels (non-filled arrows). Both fallopian tubes were visualized (filled arrows).

A 41‐year‐old female presented to our department for primary infertility evaluation. The patient did not report any previous history of an interventional procedure or major surgery. HSG was scheduled during the pre-ovulation period of the menstrual cycle to ensure there was no pregnancy. Informed consent was obtained after all possible adverse outcomes were thoroughly discussed with the patient by the experienced radiologist performing the HSG. The speculum was inserted to display the cervix, and topical anesthesia was applied. Hydrosoluble iodinated contrast media was injected after the cannula was properly positioned. Initial imaging revealed a normal‐shaped fundal cavity contour, a relatively normal fallopian tube on the left, and an ill-defined one on the right (Figure 1).

Post uterus recognition, after the delineation of the left fallopian tube, a small part of the middle segment (ampulla) of the right fallopian tube was depicted.

**Figure 2:**
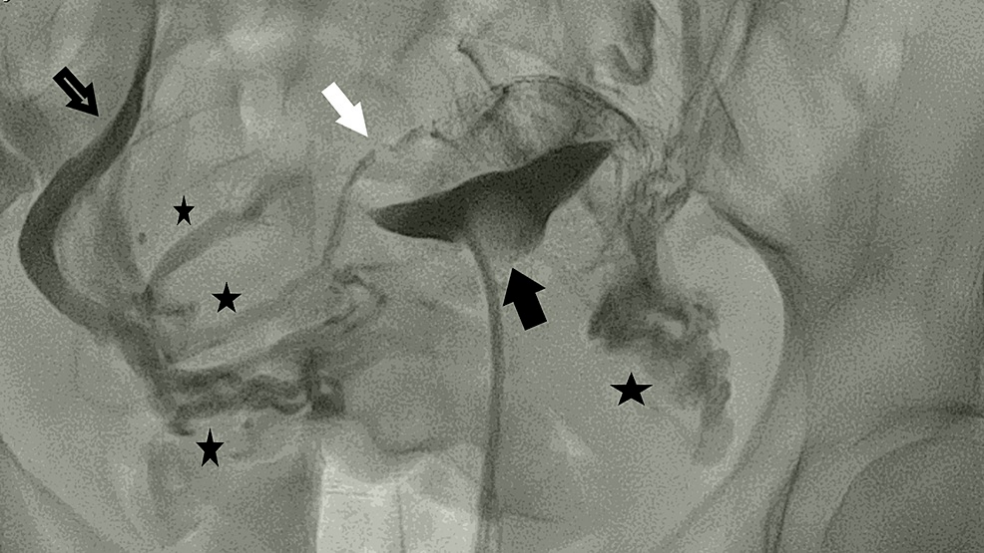
Unexpected venography. Opacified uterine cavity with normal contour (black filled arrow) and unexpected depiction of myometrial veins (white filled arrow) as well as the pelvic veins (stars), draining to the external iliac vein (outlined arrow). A balloon catheter was used to reduce pain during the HSG procedure.

The most impressive imaging finding was the introduction of contrast medium from the uterine cavity, through the myometrium, and into the draining pelvic venous system. More specifically, the myometrial vessels were opacified first, followed by the uterine vein, the internal iliac vein, and the common iliac vein subsequently (Figure 2).

Our patient did not present any allergic reactions or discomfort during and post HSG. Based on imaging findings, venous intravasation level 2 was established as a diagnosis [6].

## Discussion

We present a case of VI during HSG in a 41-year-old female. Although HSG is considered a safe procedure and VI is a rare entity (prevalence reported to vary between 0.4% and 6.9%) [7-10], the prevention of the latter could be affected by a number of predisposing factors which increase its incidence. For example, endometriosis, menometrorrhagia, primary sclerosing cholangitis (PCS), vaginal itching, and nonspecific pelvic pain are considered as predisposing factors for VI, among others [6]. On the contrary, intravasation may indirectly indicate tubal occlusion [4].

Compared to lipo-soluble contrast media, water-soluble ones are associated with decreased complications’ incidence and better imaging quality; however, in the case of VI, the correlations mentioned above were insignificant [8, 9].

The main imaging finding of VI is the opacification of myometrial vessels in a fine lace-like pattern. Subsequently, the contrast enters the larger pelvic veins and is washed out. Therefore, it is essential to distinguish VI from free intraperitoneal contrast spillage (e.g., from patent fallopian tubes), which is the desirable imaging finding and which appears as amorphous extra-uterine contrast accumulation(s) without evidence of wash-out. Intraperitoneal contrast spillage can occur due to uterine perforation caused by a defected uterine wall and will be displayed in delayed images.

VI has been well described as a phenomenon classified as either pre-procedural or procedural [6]. However, its pathobiological mechanism is yet to be fully understood. Elevated intrauterine pressure caused by either tubal occlusion or forceful contrast-medium injection is the most often proposed mechanism. From a biological perspective, the phase of menstruation or a traumatized endometrium could lead to VI [11]. The patient did not experience any discomfort or pain in our case, which contradicts current literature data, as HSG with intravasation is associated with procedural pain, with the latter potentially caused by the contrast-medium application and/or cervical fixation [5].

Although some studies have proposed that ultrasound may be useful in confirming intraperitoneal spillage, it is still to establish a minimum contrast volume level that will differentiate the former from physiologic fluid.

## Conclusions

VI during HSG should be carefully assessed to minimize severe complications. The correct type of contrast medium, proper scheduling during ovulation (mid-follicular period), and the number of predisposing factors significantly reduce intravasation incidence. At the same time, HSG’s accuracy and efficiency should be preserved to the patient’s benefit, with experienced radiologists performing, assessing, and interpreting the procedure.
